# Double-Layer Flexible Neural Probe With Closely Spaced Electrodes for High-Density *in vivo* Brain Recordings

**DOI:** 10.3389/fnins.2021.663174

**Published:** 2021-06-15

**Authors:** Sara Pimenta, José A. Rodrigues, Francisca Machado, João F. Ribeiro, Marino J. Maciel, Oleksandr Bondarchuk, Patricia Monteiro, João Gaspar, José H. Correia, Luis Jacinto

**Affiliations:** ^1^CMEMS-UMinho, Department of Industrial Electronics, University of Minho, Guimarães, Portugal; ^2^Life and Health Sciences Research Institute (ICVS), School of Medicine, University of Minho, Braga, Portugal; ^3^ICVS/3B’s–PT Government Associate Laboratory, Braga/Guimarães, Portugal; ^4^International Iberian Nanotechnology Laboratory (INL), Braga, Portugal

**Keywords:** flexible polyimide probe, polymer microfabrication, lithography processes, gold electrodes, electrophysiology, optogenetics, neuronal activity

## Abstract

Flexible polymer neural probes are an attractive emerging approach for invasive brain recordings, given that they can minimize the risks of brain damage or glial scaring. However, densely packed electrode sites, which can facilitate neuronal data analysis, are not widely available in flexible probes. Here, we present a new flexible polyimide neural probe, based on standard and low-cost lithography processes, which has 32 closely spaced 10 μm diameter gold electrode sites at two different depths from the probe surface arranged in a matrix, with inter-site distances of only 5 μm. The double-layer design and fabrication approach implemented also provides additional stiffening just sufficient to prevent probe buckling during brain insertion. This approach avoids typical laborious augmentation strategies used to increase flexible probes’ mechanical rigidity while allowing a small brain insertion footprint. Chemical composition analysis and metrology of structural, mechanical, and electrical properties demonstrated the viability of this fabrication approach. Finally, *in vivo* functional assessment tests in the mouse cortex were performed as well as histological assessment of the insertion footprint, validating the biological applicability of this flexible neural probe for acquiring high quality neuronal recordings with high signal to noise ratio (SNR) and reduced acute trauma.

## Introduction

Silicon-based neural probes have taken the stage of neuronal recordings over the last decade, especially for acute applications. These probes can be fabricated through highly reproducible procedures, support many conductive electrodes and exhibit great stability (Buzsáki et al., 2015; Jun et al., 2017b; Ulyanova et al., 2019). However, due to the intrinsic stiffness of the silicon substrate, there has been a rising interest in probes that can be fabricated in flexible biocompatible substrate materials with lower mechanical mismatch between probe and brain tissue (Weltman et al., 2016). Flexible neural probes can be highly conformable, providing adaption to brain micro- and macro-motions (Moshayedi et al., 2014; Spencer et al., 2017) by reducing tissue strain (Sridharan et al., 2015). Together with probe dimensions and implantation strategy, these advantages are important for the stability of the brain implant and for improving the quality of the recorded signals, both in acute and chronic *in vivo* applications (Kozai et al., 2015). Therefore, several flexible neural probes based on polymer substrates such as polyimide, SU-8 or Parylene C has been recently proposed with various designs (Weltman et al., 2016; Fan et al., 2020).

Nevertheless, integration of closely spaced electrode sites for high-density brain recordings, with minimal probe thickness and width, has not been widely available in flexible neural probes. Densely-packed electrode sites can facilitate neuronal data analysis by allowing spatial over-representation of neuronal activity across several sites (Scholvin et al., 2016). Electrode site size and density in neural probes is typically limited by the space needed to implement the vias that connect the electrode sites to the contact pads, while maintaining the size of the probe as small as possible. Although, the implementation of high-density neural probes with reduced dimensions is possible with CMOS technology (Raducanu et al., 2017; Dimitriadis et al., 2020), the use of lithographic processes facilitates more affordable and easily disseminated fabrication. When considering optical lithography processes, although more affordable, they present lower resolution than for example electron- or ion-beam lithography, preventing patterning of sub-micron vias. On top of this, reducing the size of the electrodes or vias beyond a certain limit can increase electrode site impedance, and running vias too close to each other can increase parasitic capacitances, regardless of the fabrication method used (metal lift-off with photolithography or photolithography followed by metal etching). These factors can lead to signal distortion and low signal-to-noise (SNR) ratio thus adding to the challenge of fabricating closely spaced electrode sites through standard and low-cost lithography processes.

The desired flexibility of neural probes also creates challenges during brain insertion/implantation, since their intrinsically low mechanical rigidity may cause buckling. The unintended deformation of the probe at this stage can prevent brain penetration or result in misplacement at the target of interest (Weltman et al., 2016). To overcome these limitations various mechanical augmentation strategies that increase probes’ buckling force threshold have been applied, namely coating the probes with absorbable molecules, such as polyethylene glycol (PEG), saccharose or hydrogels, which are rigid at room temperature but dissolve when in prolonged contact with brain tissue; or the use of hard shuttle/carrier devices that help guiding the probe into the tissue and are then removed (Richter et al., 2013; Weltman et al., 2016; Lecomte et al., 2018; Na et al., 2020). But besides adding cumbersomeness to brain implantation procedures, these approaches increase the cross-sectional area of the inserted device manifold (Weltman et al., 2016), which can lead to further tissue displacement causing additional brain damage upon insertion.

Here, we present a high-density polyimide-based flexible neural probe with 32 closely spaced gold electrode sites, fabricated with standard and low-cost lithography processes, that does not require additional stiffening aids for brain insertion. The implemented double layer architecture, where intermediate metallization and polyimide passivation layers allow the integration of closely spaced electrodes at two different depths from the probe’s surface, also provides sufficient additional intrinsic stiffening to prevent buckling. With this probe we were able to successfully record *in vivo* neuronal activity from closely spaced electrode sites without resorting to any stiffening procedure for brain penetration and probe insertion. Contrarily to other flexible polymeric neural probes, by not using buckling force threshold augmentation coatings or shuttles, the insertion footprint of our probe was limited to its reduced dimensions. Before *in vivo* recordings and histological assessments, we also fully characterized the viability of this new fabrication approach by metrology of structural, mechanical and electrical properties as well as chemical composition, using scanning electron microscopy (SEM), compression tests, energy-dispersive x-ray spectroscopy (EDS), x-ray photoelectron spectroscopy (XPS) and electrochemical impedance spectroscopy (EIS). Given that the proposed fabrication process employs only standard lithography techniques, multiple probe geometries with different arrangements of closely spaced electrode sites can be easily achieved at low-cost while maintaining structural properties.

## Materials and Methods

### Probe Fabrication

**FIGURE 1:**
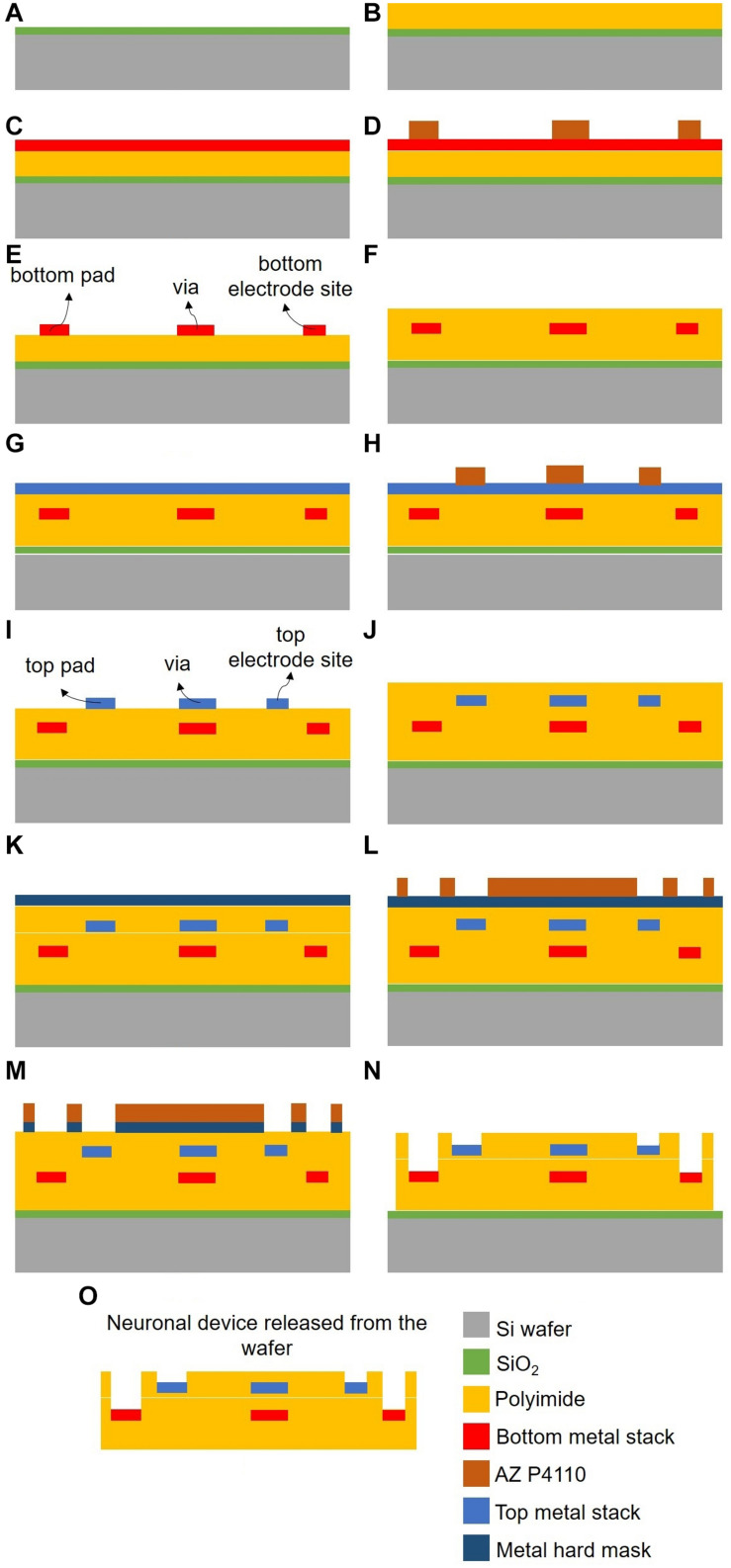
Neural probe fabrication process (not to scale). Microfabrication processes for: polyimide substrate deposition **(A–B)**; patterning and passivation of bottom **(C–F)** and top **(G–J)** electrode sites, vias and contact pads; final polyimide etching to expose electrode sites and define probe geometry **(K–N)**; and probe release **(O)**.

A standard photolithography process was employed, alternating polyimide and metallization layers to pattern the electrode sites, vias, and contact pads. Figure 1 shows a graphical depiction of the probe fabrication steps.

A sacrificial film (0.5 μm thick SiO_2_) was initially deposited by chemical vapor deposition (CVD) over a 200 mm silicon wafer to allow detachment of the probe at the end of processing (Figure 1A). This was followed by spin-coating, soft-baking, and curing a layer of non-photodefineable polyimide (PI-2611 from HD MicroSystems), approximately 7.5 μm thick, which formed the base of the probe (Figure 1B). Then, a metal stack [TiW/AlSiCu/TiW (50/500/50 nm), Au (200 nm), Cr (10 nm), and AlSiCu/TiW (250/50 nm)] was sequentially sputtered (Figure 1C). The electrode sites, vias and contact pads of what would become the bottom layer were then defined by UV photolithography with a Cr hard mask and photoresist (AZ P4110 from MicroChemicals), and patterned by reactive ion etching (RIE), followed by O_2_ stripping to remove the remaining photoresist (Figures 1D,E). A second 7.5 μm thick layer of PI-2611 was again spin-coated, baked and cured to ensure passivation of the bottom metal layer (Figure 1F). A second metal stack [TiW/AlSiCu/TiW (50/500/50 nm), Au (200 nm), Cr (10 nm) and AlSiCu/TiW (350/50 nm)] was sputtered sequentially (Figure 1G). The electrode sites, vias and contact pads of the top layer were defined by UV photolithography with direct laser writing laser (DLW) and photoresist (AZ P4110 from MicroChemicals), and patterned by RIE, followed by O_2_ stripping to remove the remaining photoresist (Figures 1H,I). A final layer of PI-2611 was spin-coated, soft-baking and cured to ensure passivation of the probe (Figure 1J). A metal hard mask [AlSiCu/TiW (600/50 nm)] was sputtered (Figure 1K), defined by UV photolithography with a Cr hard mask and photoresist (AZ P4110 from MicroChemicals), and patterned with RIE (Figures 1L,M). Then, an O_2_/CF_4_ etching process was performed for PI-2611 etching, exposing the electrode sites and contact pads of both the bottom and top electrode layers (Figure 1N). Finally, two wet etching processes (AlSiCu and Cr etching) and an HF vapor etching process (SiO_2_ etching) were performed to remove the hard mask, to expose the Au in the electrode sites and contact pads and to release the neural probe from the silicon wafer (Figure 1O). The metal stacks used in the fabrication process are well-established standard metal stacks for lithographic processes, ensuring good conductivity for recording neuronal signals.

### Probe Packaging

The released neural probes were packaged with a custom-designed printed circuit board (PCB) to allow access to the electrodes through an headstage (RHD2132, Intan), which includes a pre-amplifier and a digitizer, using an omnetics connector (A79022-001). An FPC connector (FH29DJ-50S-0.2SHW, Hirose Electric Co.) connected the flexible contact pads on the probe side to the PCB. The PCB package allowed the electrode site gold surface cleaning procedure, signal acquisition for impedance measurements and *in vivo* recordings.

In order to obtain clean gold surfaces at the electrode sites, removing unwanted residuals from the fabrication process, the tip containing the electrode sites was immersed in a solution of 50 mM potassium hydroxide (KOH) and 25% of hydrogen peroxide (H_2_O_2_) for 10 min, as described in Fischer et al. (2009), followed by a 50 mM KOH sweep, using a potentiostat (Reference 600, Gamry Instruments) and a sweep from −200 to −1,200 mV (vs. Ag/AgCl) at 50 mV/s scan rate.

### Probe Characterization

Scanning electron microscopy measurements were performed to structurally evaluate the fabricated probe using a NanoSEM – FEI Nova 200 equipment, at SEMAT (University of Minho).

Energy-dispersive x-ray spectroscopy was performed to chemically evaluate the neural probe and confirm that the electrode sites were etched correctly to expose the gold surface. EDS was performed during SEM measurements, using an EDAX - Pegasus X4M equipment. XPS was also performed to chemically evaluate the surface of the probe at a depth of approximately 10 nm. XPS measurements of Au and Ce atomic concentrations were performed using a Thermo Scientific Escalab 250 Xi equipment, and considering an analysis area of 650 μm diameter, as a function of the etching time.

Electrochemical impedance spectroscopy measurements were performed with a potentiostat (Reference 600, Gamry Instruments) in a three-electrode configuration in 0.9% saline electrolyte: working electrode (each electrode site of the probe), counter electrode (Pt wire) and reference electrode (Ag/AgCl). An AC sinusoidal signal of 5 mV with a frequency between 100 Hz and 10 kHz was applied, and the impedance value at 1 kHz for each electrode site was recorded.

Longitudinal tensile stress and compressional tests were performed with the fabricated neural probe to assess its mechanical robustness. In the tensile trials, a tensile load was applied until failure, and the resulting extension was measured. The crosshead extension rate was set at 0.2 mm/min. Young’s Modulus was determined by taking the mean slope of the linear elastic region of the stress-strain curve. For longitudinal compression tests, the neural probe was fixed perpendicularly to the dynamometer rigid base surface, moving at a constant rate of 5 mm/min. Finally, to measure the forces required to implant and extract the probe in a brain phantom, 0.6% agar gel was used as previously reported (Das et al., 2007; Pomfret et al., 2013). The neural probe was fixed to the dynamometer perpendicular to the agar gel surface, moving toward it at constant rate of 5 mm/min. Traditionally, insertion speeds range from slower at around 600 μm/min to as high as 60 mm/min (Weltman et al., 2016). After the probe’s shaft fully penetrated the agar, it stopped for 30 s before the extraction tests began. All tests were carried on a Shimadzu AG-IS dynamometer equipped with a 10 N load cell.

### Electrophysiology and Optogenetics

Experiments were conducted in accordance with European Union Directive 2016/63/EU and the Portuguese law on the protection of animals for scientific purposes (DL No 113/2013). This study was approved by and the Portuguese National Authority for Animal Health (DGAV, 8519) and the Ethics Subcommittee for Life and Health Sciences of University of Minho (SECVS, 01/18).

Emx1-Cre; Ai27D male mice (*n* = 4), which express a modified form of channelrhodopsin in cortical pyramidal neurons, were used in this study. Mice were obtained by crossing homozygous Emx1-IRES-cre mice with homozygous Ai27D mice (JAX stocks #005628, #012567) (Gorski et al., 2002; Madisen et al., 2012). Briefly, mice were anesthetized by an intraperitoneal injection of ketamine (75 mg/kg) and medetomidine (1 mg/kg) mix. Following positioning in a stereotaxic frame (Stoelting), a midline skin incision across the top of the head exposed the skull, in which a burr hole above the motor cortex (M1) was opened [1.5 mm AP and 1.5 mm ML from bregma, according to Franklin and Paxinos (2007)]. After careful removal of the dura of the exposed surface of the brain, the neural probe, connected to the PCB and attached to a precision micrometric stereotaxic arm (1760, Kopf Instruments), was lowered to at least 250 μm from the brain surface. A stainless-steel screw on the back of the skull was used as ground. Extracellular signals were acquired at 30 KS/s with an Open Ephys acquisition system (Siegle et al., 2017).

To evoke neuronal activity from M1 neurons expressing light-gated channelrhodopsin, blue light pulses (473 nm wavelength, 5 Hz with, 50 ms pulse width) were delivered to the surface of the brain (M1, same coordinates as above) by an optical fiber (0.22 NA, 200 μm core, FG200LEA, Thorlabs), held by a micromanipulator, and connected to a DPSS laser source (CNI). A waveform generator (DG1022, Rigol) connected to the laser source performed the pulse triggering.

Signals were analyzed with custom Matlab code (Mathworks) and JRClust (Jun et al., 2017a). Extracellular recordings were filtered between 0.3 and 6 kHz and spikes were detected using an amplitude threshold at least five times higher than an estimate of background noise standard deviation (Quiroga et al., 2004). Initial spike sorting, where spikes were assigned to different unit clusters, was automatically performed by JRClust, followed by manual cluster curation based on visual inspection of spike waveforms, inter-spike interval distribution and auto-/cross-correlograms.

### Histology

To determine neural probe localization during recordings and measure its acute insertion footprint, probes were coated in DiI stain (1, 1′-Dioctadecyl-3,3,3′,3′-Tetramethylindocarbocyanine Perchlorate) (D3911, ThermoFisher) before brain insertion. This lipophilic red fluorescent dye has been extensively used for neuronal tracing and more recently to determine neural probes positioning post-implantation (Juavinett et al., 2019; Shin et al., 2019). A C57BL/6 mouse was used for the insertion footprint experiment. The DiI coated neural probe was inserted into the cortex of the animal to a depth of 500 μm.

At the end of the experiments, animals were transcardially perfused with 0.9% saline followed by 4% paraformaldehyde (PFA) in Phosphate Buffer Saline (PBS) and the brain was carefully removed and stored in 4% PFA overnight. Brains from the neural recordings were sectioned coronally at 80 μm in a vibratome (VT 1000S, Leica). The brain for the insertion footprint test was sectioned horizontally at 30 μm in a cryostat (CM 1900, Leica). All slices were counterstained with DAPI nucleic acid stain (1:1000) (A1001, PanReac AppliChem). Images of mounted sections were acquired with a confocal fluorescence microscope (FV3000, Olympus).

## Results and Discussion

### Fabricated Flexible Neural Probe

**FIGURE 2:**
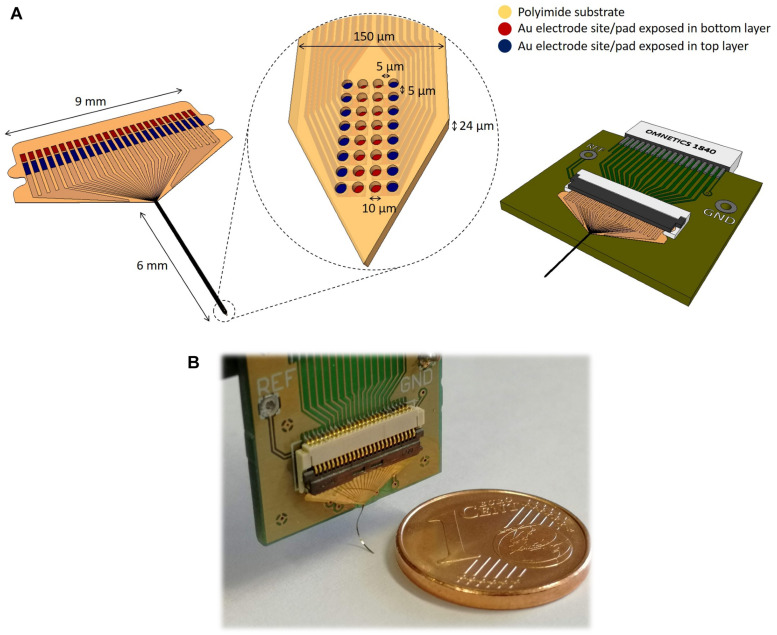
Schematic illustration of the flexible polyimide neural probe design and its dimensions. **(A)** Full probe view including flexible connector pad (left) and printed circuit board (PCB) for packaging (right). The central inset shows the distribution of the closely spaced electrode sites at the tip of the probe. **(B)** Fabricated flexible neural probe coupled to the PCB.

Figure 2 shows the design of the double-layer polyimide flexible probe with closely spaced electrode sites. The neural probe contains 32 circular gold electrode sites, each one with approximately 10 μm diameter. The inter-site distance is 5 μm in both directions, creating a closely spaced matrix of electrode sites. The neural probe has approximately 150 μm width, 24 μm thickness and 6 mm shaft length. In order to fabricate a high-density probe with closely spaced electrode sites in a flexible substrate, a two-layer design with electrode sites at two different depths (top and bottom) from the probe surface (6 and 12 μm, respectively) was implemented. Electrodes, vias, and contacts were distributed across two different depths and patterned in sequential deposition, photolithographic and RIE steps. This strategy allows the electrode sites to be in close proximity and still have enough space for running the vias to the contact pads along the two layers, while keeping the probe with a small physical footprint. Implemented line width was of 4 μm with a line spacing of 4 μm. Considering the entire fabrication process and the several lithographies on polyimide layers, the resolution of the process was at its limit. Polyimide was chosen as the substrate due to its superior stability, biocompatibility, and low elastic modulus (Rubehn and Stieglitz, 2010; Weltman et al., 2016). Additionally, the use of non-photodefinable polyimide permitted the implementation of low-cost etching fabrication procedures.

**FIGURE 3:**
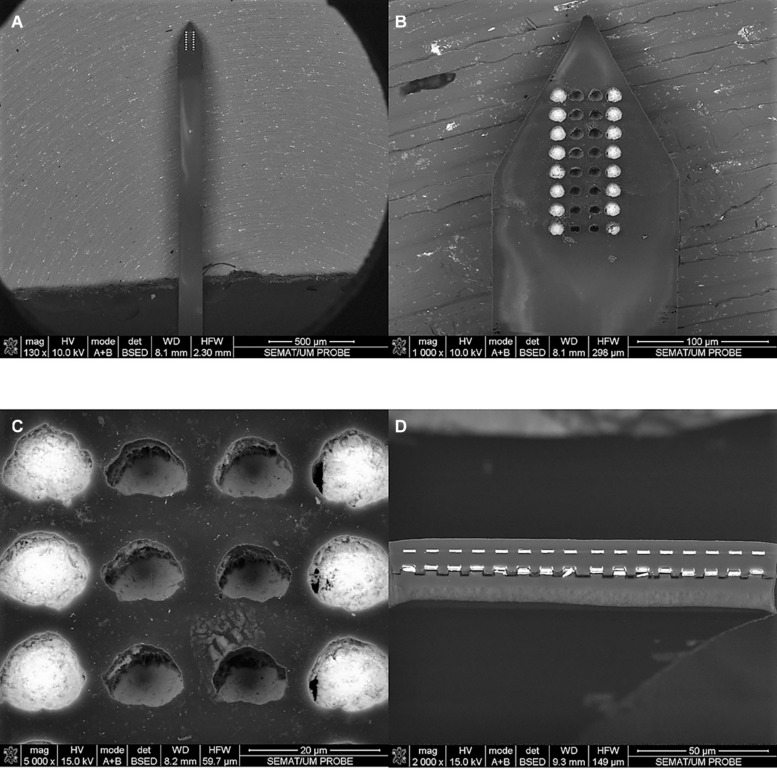
SEM images of the flexible polyimide neural probe. **(A)** top view of probe shaft and tip containing the electrode sites. **(B,C)** detail view of the probe tip, showing the closely spaced electrode sites at two different depths; and **(D)** cross-section view (obtained with a scalpel) of the probe shaft showing two metallic vias separated by the intermediate polyimide layer.

Figure 3 shows SEM images of a fabricated probe with the exposed gold surface of the electrode sites at two different depths. The observed clear color difference between top and bottom electrode sites due to their different depths reveals correct polyimide passivation (Figures 3A–C). However, the electrode sites have irregular exposed areas and the top electrode sites appear to have a slightly higher exposed area when compared with the bottom electrode sites (Figure 3C). This is most likely due to the polyimide etching process, which was a continuous process that sequentially exposed the top electrodes first (one layer of polyimide etched), then the bottom electrodes (two layers of polyimide etched), and finally defined the neural probe geometry (three layers of polyimide etched). Thus, until the removal of the third layer of polyimide, electrode sites already exposed were subjected to the etching process which increased the probability of polyimide lateral over-etching (and this probability was higher in top than in bottom electrode sites due to the sequential etching process). Despite the difference in exposed surface, the precision of alignment between top and bottom electrode sites is maintained and benefited from the use of DLW to pattern the second metal layer which allowed a higher resolution. Figure 3D shows the bottom and top metal tracks patterned along the polyimide substrate and the absence of short circuit between these two metallic layers, separated by an intermediate polyimide layer.

### Chemical and Electrical Characterization of Neural Probe

To assess the viability of the new fabrication process, including the successful exposure of the gold electrode sites through the various stages of polyimide etching, metrology of chemical composition analysis and electrical properties and were performed by EDS, XPS, and EIS.

**FIGURE 4:**
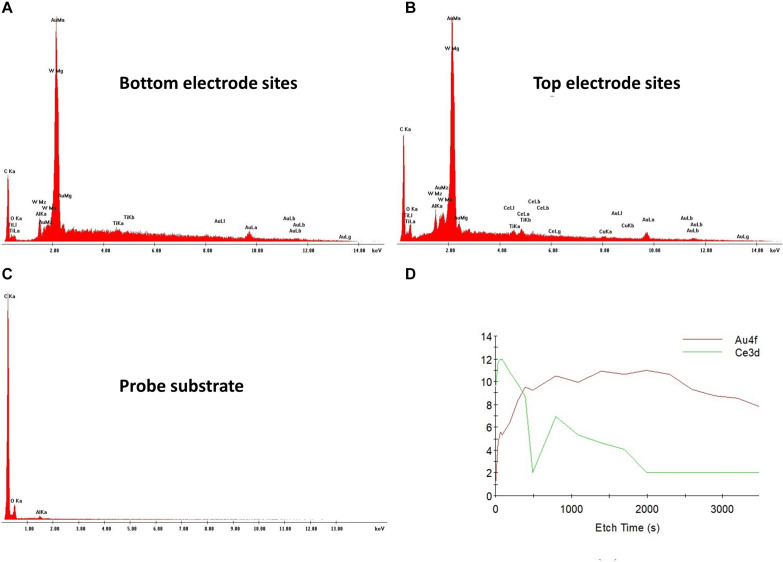
EDS and XPS analysis of the neural probe. Chemical composition of a bottom **(A)** and a top **(B)** electrode site with clear peaks for gold. **(C)** Chemical composition of the probe substrate with a clear peak for carbon. **(D)** XPS results for the probe surface showing an increase of gold as function of the etching time.

Figure 4 shows the EDS and XPS results. EDS analysis revealed gold (Au) as the most prevalent element at the bottom and top electrode sites (Figures 4A,B respectively) with a percentage by mass higher than 56 wt%, confirming successful etching during fabrication. Other detected elements include carbon (C) (higher than 24 wt%), tungsten (W) (higher than 3 wt%) and aluminum (Al), titanium (Ti), oxygen (O), copper (Cu), and Cerium (Ce) (all below 3 wt%). The presence of other elements in lower concentrations is expected, as C is in the polyimide layers, Al, Ti, Cu, and W are part of the metal stacks and Ce and O are residuals of the wet and polyimide etching processes. The EDS analysis detected the chemical element C as being the most prevalent in the probe substrate (approximately 80 wt%) (Figure 4C), as expected from the presence of a carbonyl group in the polyimide polymer film. XPS measurements further validated the presence of Au at the electrode sites surface. This analysis showed that the atomic concentration of Au at the probe surface increased as a function of etching time, while Ce decreased (Figure 4D), further confirming successful etching processes during the fabrication of the flexible probe.

**FIGURE 5:**
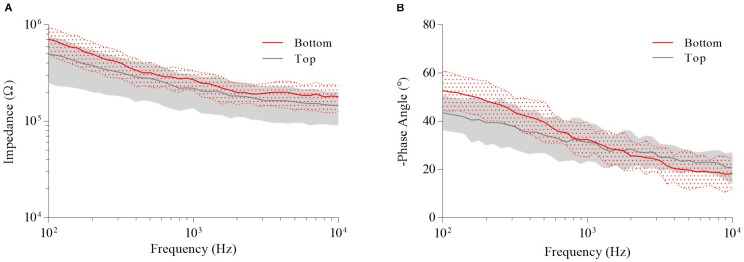
Electrochemical impedance spectroscopy (EIS) measurements of the bottom and top electrode sites of fabricated neural probe. **(A)** Mean impedance vs. frequency. **(B)** Mean phase angle vs. frequency.

Following cleaning of the electrode sites with a potassium hydroxide (KOH) and hydrogen peroxide (H_2_O_2_) solution to obtain clean gold surfaces (Fischer et al., 2009), EIS measurements were performed. Figure 5 shows the mean impedance vs. frequency and the phase angle vs. frequency of gold electrodes of the neural probe. The mean impedance magnitude of the bottom and top electrode sites at 1 kHz was 268 ± 63 kΩ and 214 ± 83 kΩ, respectively. The mean phase angle at 1 kHz was −32.5 ± 7.4° and −31.2 ± 7.8°, respectively. The gold electrodes mean impedance is low considering the 10 μm diameter electrode area, which may have been potentiated by the fabrication process, specifically the polyimide etching process. As previously mentioned, the polyimide etching was a continuous process in which electrode sites already exposed were subjected to the etching process. This may have led to an increase of the electrodes surface roughness and consequent reduction in electrode impedance. Kim et al. (2014) reported grain structures that increased electrodes surface area, resulting in impedances of 126 kΩ at 1 kHz for 10 μm sized gold microelectrodes. Thus, the benefits of the potentially increased electrodes surface roughness allowed that our 10 μm diameter electrode sites reached an impedance within the optimal range for neuronal recordings with low intrinsic noise (below 15 μV RMS *in vivo*) and high signal to noise ratio (SNR) (over 5) (Scholvin et al., 2016; Neto et al., 2018), without the need for any additional electrodeposition steps.

### Mechanical Characterization of Neural Probe

**FIGURE 6:**
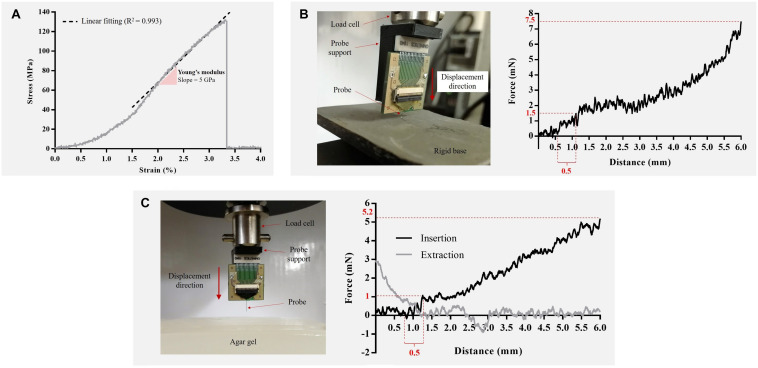
Mechanical characterization of fabricated neural probe. **(A)** Stress-strain curve. The Young’s modulus, determined by the slope of the linear region of the stress-strain curve, was approximately 5 GPa. **(B)** Setup arrangement (left) and the force vs. displacement plot (right) for the longitudinal compression tests on a rigid base. The maximum compression force was 7.5 mN at the maximum displacement. **(C)** Setup arrangement (left) and the force vs. displacement plots (right) for insertion and extraction tests on brain phantom (0.6% agar gel). The minimum force required for probe insertion was approximately 1 mN (dimpling of 0.5 mm). The maximum implantation force was 5.2 mN at the maximum displacement (6 mm).

Tensile tests on neural probe specimens revealed a Young’s modulus of 5 GPa, as shown in Figure 6A by the obtained stress-strain curve. This ensured that the probe was flexible, with a stiffness of at least an order of 30 lower than a silicon probe with the same dimensions (Lecomte et al., 2018), and closer to 7 kPa Young’s modulus of the mouse brain cortex (Weltman et al., 2016).

Figure 6B shows the setup and the force vs. displacement plot for the longitudinal compression tests on a rigid base. The neural probe was compressed without breaking to a total traveling distance of 6 mm which equals the length of the probe. The results showed an increase of the force as the neural probe was compressed. At the maximum displacement (6 mm), the observed compressed force was of 7.5 mN.

The insertion and extraction experiments showed that the fabricated neural probe could readily penetrate a brain phantom (0.6% agar gel) without buckling and travel a length of 6 mm inside the gel, as shown on the force vs. displacement plots in Figure 6C. Insertion test revealed that the minimum force required for probe insertion (insertion force) is approximately 1 mN (dimpling of 0.5 mm). This is in accordance with the theoretical insertion force necessary for neural probe insertion into a rodent brain (Sharp et al., 2009; Wester et al., 2009). After penetration, the insertion force increased due to the frictional forces of the agar gel and the force required for the probe to continue the path on the medium. The maximum implantation force (5.2 mN) was obtained at the maximum displacement (6 mm). The force required to extract the neural probe was maximum (3 mN) when the probe was fully inserted and rapidly decreased to zero as the probe moved outward from the agar gel.

Longitudinal forces play a key role while implanting devices into the brain (Goncalves et al., 2018). The mechanical tests performed show that the fabricated neural probe can be inserted into the brain without additional insertion aids to augment is buckling force threshold, since the compression force of the neural probe on a rigid base at 0.5 mm of displacement (1.5 mN) was greater than the insertion force of the neural probe in agar (1 mN). The forces involved in these experimental tests are in accordance with those reported for polymeric neural probes (Haj Hosseini et al., 2007; Singh et al., 2016; Smith, 2019).

### *In vivo* Electrophysiology

**FIGURE 7:**
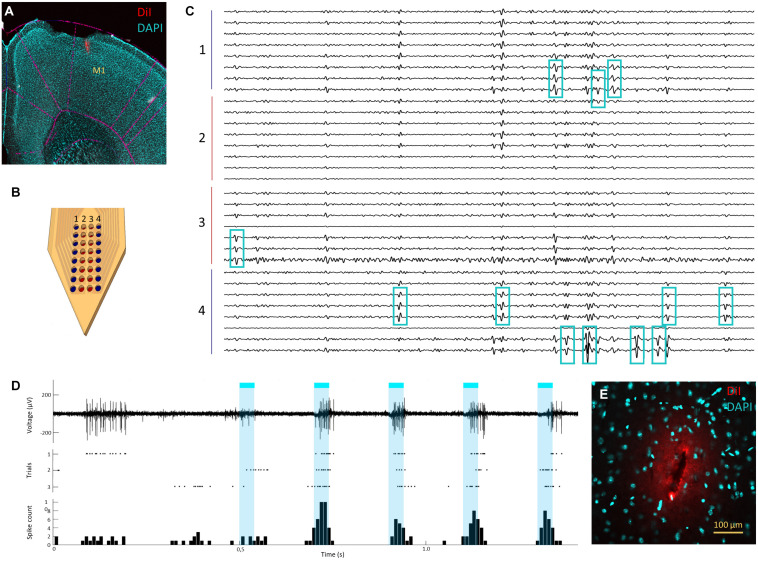
*In vivo* recordings in mouse cortex and histological assessment of insertion footprint. **(A)** Neural probe insertion track (red) in primary motor cortex (M1) (magnification 2×). **(B)** Neural probe electrode site layout with top electrode sites (columns 1 and 4) in blue and bottom electrode sites (columns 2 and 3) in red. **(C)** Example of spontaneous neuronal activity recorded simultaneously from all top (groups 1 and 4) and bottom (groups 2 and 3) electrode sites of the neural probe (signals band-pass filtered between 0.3 and 6 kHz). Blue squares mark the three channels where twelve isolated single units display the highest signal amplitudes. **(D)** Spontaneous and opto-evoked neuronal activity with light stimulation periods marked in blue; an example signal trace showing spontaneous neuronal activity (0–0.5 s) and opto modulation trials (0.5–1.5 s, light ON for 5 ms marked in blue) (top; filtered 0.3–6 kHz), spike raster plot for three opto-stimulation periods, each with five trials (middle) and cumulative peri-stimulus time histogram (PSTH) with total spike count across time for trials over three stimulation periods (bottom). **(E)** neural probe insertion footprint in cortex on horizontal brain slice (magnification 20×); measured footprint was 135 μm wide and 26 μm thick, which approx. matches the neural probe’s dimensions.

To perform functional assessment of the closely spaced electrode sites in the probe, electrophysiological brain recordings were performed *in vivo*. Figure 7 shows spontaneous and opto-evoked neuronal activity recorded from the primary motor cortex (M1) of anesthetized mice. After removing dura and exposing the brain surface, it was possible to easily insert our flexible probe in M1 (Figure 7A) without having to resort to any insertion aid, as predicted by the mechanical tests performed.

Figures 7B,C shows an example of recorded neuronal activity across all channels in the probe, where it is possible to observe multiple spiking units across several recording sites. The average RMS from the signal across all channels was approximately 11 μV, with isolated spikes crossing a threshold of at least five times that value. The closely spaced configuration design allowed the recording of the same neuronal activity from the same neurons across several electrode sites, which allowed reliable spike sorting with a conventional spike sorting algorithm. From the test electrophysiology recordings performed with these probes an average of 15 ± 3 single units per recording (average of four recordings) were isolated. Figures 7B,C shows a segment of neuronal activity traces where 12 isolated single units can be observed. Although each unit is usually detectable across several electrode sites, both top and bottom, because bottom electrode sites are more likely to sit further away from neurons generating the signals, they also tend to record smaller amplitude signals. Nevertheless, it is still possible to isolate units that have higher signal amplitudes on bottom electrode sites or that occur exclusively on these sites, although in a significantly smaller proportion (approximately 10%) than on top electrode sites. Despite the smaller signal amplitude and a smaller proportion of isolated single units on bottom electrode sites, these can help to disambiguate signals recorded with higher amplitudes on top electrode sites, thus facilitating spike sorting. Spike sorting can be facilitated by spatial over-representation of neuronal activity across several recording sites (Scholvin et al., 2016; Jun et al., 2017b), especially in recording devices where the electrode geometry is fixed. Spike sorting algorithms depend on the co-occurrence of spikes generated by each neuron on different electrodes sites to allow disambiguation based on the magnitude of the signal as a function of distance to each of these sites (Rey et al., 2015; Jun et al., 2017a). However, neurons that are very close together may have negligible signal magnitude differences at the few electrode sites in their vicinity (and these differences are reduced the further away neurons are from the electrode sites due to global signal attenuation), which may hinder their isolation into separate units. Thus, having the same spike represented simultaneously in many electrode sites can potentially facilitate finer disambiguation by allowing spatial oversampling of the same signal and detection of finer differences in signal magnitude. Such layout is especially beneficial for improving the quality of spike sorting in neuronal recordings from dense laminar structures in the brain, such as the hippocampus or the cortex, where neurons can be tightly packed in layers.

To further assess the capability of the neural probe in detecting modulated neuronal activity and performance in a scenario of an *in vivo* optogenetics experiment, a typical application sought after by many experimental neuroscience labs, neuronal responses were evoked from cortical neurons expressing channelrhodopsin by shining blue light into the cortical region. Figure 7D shows successful brain recordings, with high SNR, of both spontaneous and opto-evoked neuronal activity. Although no apparent optoelectrical artifacts were observed during light stimulation, a brief (<10 ms) and small (<30 μV) deflection of baseline activity was observed at the start of each stimulation pulse. Considering that optical stimulation was being delivered at brain surface, this effect may be due to opto-induced activity of cortical neurons further away from the recording site.

### Insertion Footprint Assessment

The insertion footprint, i.e., the size of the hole resulting from the acute neural probe insertion into the brain was measured in horizontal slices and is shown of Figure 7E. The measured footprint of a cortical insertion was approx. 135 by 26 μm wide which is close to the probe’s maximum width and thickness. The opening angle of the probe below the electrode sites is of approx. 30° which may have contributed to dispersion of the penetration force and reduced tissue displacement, keeping the insertion footprint close to the probe’s maximum dimensions.

Other polymeric flexible neural probes that require coatings with absorbable molecules to increase their buckling force threshold to permit successful brain implantation have reported increases in the cross-section of the probe ranging from 6 to 1,500 times the original dimensions (Weltman et al., 2016). For example Xiang et al. (2014) reported a flexible polyimide neural probe with a higher width (200 μm) and lower thickness (10 μm) than ours, although it required coating with maltose for successful brain insertion. The application of the coating increased both the width and thickness of the probe to an additional 200 μm (to a total of 400 μm width and 220 μm thickness) for the buckling threshold to surpass the force for brain insertion, which would lead to a larger insertion footprint. Additionally, as in other neural probes using coatings for stiffness augmentation, besides the time-consuming and delicate application of the coating, it was necessary to wait a for the maltose to be absorbed. Dissolution rates of coatings may range from a few minutes up to a few days, depending on the coating material used (Weltman et al., 2016). By avoiding the use of any coating, and the necessary waiting time for coating dissolution/absorption, our neural probe permits reduced acute trauma and faster acute electrophyiological experimental protocols.

Flexible neural probes that have a smaller cross-sectional area (below 10 μm) typically require shuttle/carrier devices to guide the neural probe into the tissue. These structural shuttles are usually fabricated in hard materials such as silicon or metals including tungsten or stainless steel. For example, Kim et al. (2013) presented a 10 μm thick Parylene C neural probe that required an attached 250 μm tungsten wire for brain insertion which led to implant footprints over 300 μm wide. Similar results were also reported for other flexible neural probes of similar dimensions using hard metal shuttles as carrier devices (Richter et al., 2013; Zhang et al., 2020). Although some of these approaches may lead to acute insertion footprints that are comparable to the ones observed with our probe, the fact that the tissue is pushed away from the probe by the carrier (then removed) may lead to poorer signal quality since larger scar tissue areas are expected and the electrode sites will theoretically lay further away from the tissue generating the neuronal signals, even when tissue re-accommodation is considered. Nevertheless, promising recent approaches using materials with tunable properties that can increase stiffness without any increase in size (Wen et al., 2019), control of flexibility by microfluidic pressure (Rezaei et al., 2019) or thinned metal wire carriers (Zhao et al., 2019) may lead to new improved solutions for implanting increasingly smaller flexible neural probes in the brain. The neural probe presented in this paper, although thicker than other recently reported flexible neural probes, removes the cumbersomeness of the implantation procedure with insertion aids and reduces the fabrication complexity, while delivering a high electrode site count per area as well as small inter-site distances and maintaining a low mechanical rigidity within the desired range for a flexible substrate neural probe.

## Conclusion

This paper presents a new flexible polyimide neural probe with closely spaced electrode sites for high-density neuronal recordings, which requires no additional coating/stiffening procedures to allow brain insertion. With the microfabrication process described here, it was possible to use standard and low-cost photolithography techniques to fabricate a double-layer high-density probe in a flexible substrate with low stiffness. The intermediate metal and polyimide passivation layers of the double layer design allowed a matrix arrangement of closely spaced electrodes while providing just sufficient additional stiffening to prevent buckling during brain insertion. The obtained size and mean impedance of the gold electrode sites facilitated *in vivo* recordings with high SNR and subsequent single-unit spike-sorting. Our results show that the probe presented here can be amenable for widespread use by removing the necessity of brain insertion aids, reducing insertion footprint, and providing high SNR neuronal activity recordings. Additionally, since only standard and low-cost lithography processes were used it can be scaled to include, for example, multiple shank designs and different electrode configurations on each shank just by simple changes to mask patterning.

## Data Availability Statement

The original contributions presented in the study are included in the article/supplementary material, further inquiries can be directed to the corresponding author/s.

## Ethics Statement

The animal study was reviewed and approved by Ethics Subcommittee for Life and Health Sciences of University of Minho (SECVS, 01/18) and Portuguese National Authority for Animal Health (DGAV, 8519).

## Author Contributions

All authors contributed to the work presented in this manuscript. SP, JFR, JG, and JHC conceived and designed the flexible neural probe. SP and JFR performed the fabrication process, supervised by JG. JAR, MJM, and OB performed probe characterization. SP and JAR analyzed the probe characterization results. LJ designed and performed the *in vivo* experiments and histological analysis. PM provided the transgenic animals and performed the optogenetic experiments with LJ. FM and LJ analyzed the neuronal activity data. SP and JAR wrote the first draft of the manuscript. JHC and LJ revised and wrote the final version of the manuscript, acquired funding and supervised all fabrication, probe characterization and animal validation works. All authors approved the final version of the manuscript.

## Conflict of Interest

The authors declare that the research was conducted in the absence of any commercial or financial relationships that could be construed as a potential conflict of interest.
